# Immediate effect of ACL kinesio taping technique on knee joint biomechanics during a drop vertical jump: a randomized crossover controlled trial

**DOI:** 10.1186/s13102-019-0144-6

**Published:** 2019-11-11

**Authors:** Weerawat Limroongreungrat, Chuanpis Boonkerd

**Affiliations:** 1 College of Sports Science and Technology, Mahidol University, Puthamonthon 4 Road, Nakhon Pathom, 73170 Thailand; 2 Department of Physical Therapy, Faculty of Allied Health Sciences, Thammasat University, Pathumthani, Thailand

**Keywords:** Kinesio tape, Knee joint, Kinetic, Kinematic, 3-D analysis

## Abstract

**Background:**

The purpose of this study was to investigate the effect of an ACL Kinesio Taping technique (ACL-KT) on knee joint biomechanics during a drop vertical jump (DVJ).

**Methods:**

Twenty healthy male participants (age 21.1±0.3 years; mass 64.2±4.3 kg; height 174.2±5.5 cm) participated in this study. The participants performed a DVJ and landed onto 2 adjacent force platforms under both ACL-KT and placebo (PT) conditions. All data were collected with 3-D motion analysis and comparison peak knee joint angles and moments, and knee joint angle at initial contact (IC) between conditions analyzed using a paired sample t-test. Statistical parametric mapping (SPM) was selected to assess difference between groups for the entire three-component knee trajectory during the contact phase.

**Results:**

ACL-KT had a significant effect on decreasing knee abduction angle at IC (1.43±2.12 deg.) compared with the PT (−1.24±2.42 deg.) (*p*=0.04). A significant difference in knee abduction angle between the taping conditions was found between 100 ms before IC, at IC and 100 ms after IC (*p*<0.05). There were no significant differences (*p*>0.05) found between conditions in any of the other variables.

**Conclusion:**

This result confirmed that the application of ACL-KT is useful to reduce knee abduction angle at IC during a DVJ in healthy participants. Therefore, ACL-KT may be an acceptable intervention to reduce ACL injury risk.

**Trial registration:**

Retrospective registered on 25 September 2018. Trial number: TCTR20180926005

## Background

Anterior cruciate ligament (ACL) injury of the knee joint leads to short-term disability [1], impaired function [2], possible loss of opportunity and osteoarthritis [3]. Over the last decade, the development of ACL injury prediction and assessment models have improved our understanding of the associated injury mechanisms and the identification of ACL injury risk factors [1], leading to the development of effective injury prevention programs [3]. The neuromuscular and biomechanical aspects related to ACL injury are modifiable risk factors, which may be adjusted by an applied intervention [1, 3] to reduce the risk of ACL injury [1]. A measure of high knee abduction angle, and moment, during a drop vertical jump (DVJ) predicts an ACL injury with high specificity and sensitivity [4] and has a high reliability to screen lower limb injury [5]. Therefore, biomechanical assessment of a DVJ may be used to determine the efficacy of an ACL intervention on injury risk.

Kinesio tape (KT) is an elastic therapeutic tape, which is proposed to be beneficial [6] in the prevention and treatment of sports injury [7] by decreasing pain [8] and increasing proprioception [9], muscle activity [10] and active ROM [11]. The ACL Kinesio Taping technique (ACL-KT) is a taping method used to prevent ACL injury via application of the tape onto the tibia in an anteroposterior direction. Importantly, no studies have focused on the issue of ACL-KT through the generation of biomechanical changes, with further research required to bolster our knowledge on the potentially beneficial effects of ACL-KT application.

Understanding the effect of KT on biomechanical changes during movements associated with an ACL injury is important since it may help the physiotherapist, athletic trainer or individual to choose an effective injury prevention. Thus, the present study was aimed at investigating the effects of ACL-KT on knee joint mechanics during a DVJ using a 3D motion analysis system. We hypothesized that ACL-KT would reduce the associated movement risk of an ACL injury in healthy subjects.

## Methods

### Participants

Twenty healthy male participants (*mean*±*SD*: age, 21.1±0.3 years; mass, 64.2±4.3 kg; height, 174.2±5.5 cm) participated in this study. Participants had no history of lower limb injury within the previous six months and were excluded from the study if they previously had experienced an ACL injury. All subjects were familiarized with the experimental procedure and associated risks and provided their written informed consent to participate. The study was approved by the Mahidol University Institutional Review Board.

Using data from the literature [12], sample size was estimated for a power of 0.8 and alpha level of 0.5. The sample size was determined by GPower 3 Software (version 3.1.9.2) using the F test for repeated measures ANOVA [13]. 20 subjects were deemed adequate.

### Instrumentation and biomechanical model

**Fig. 1:**
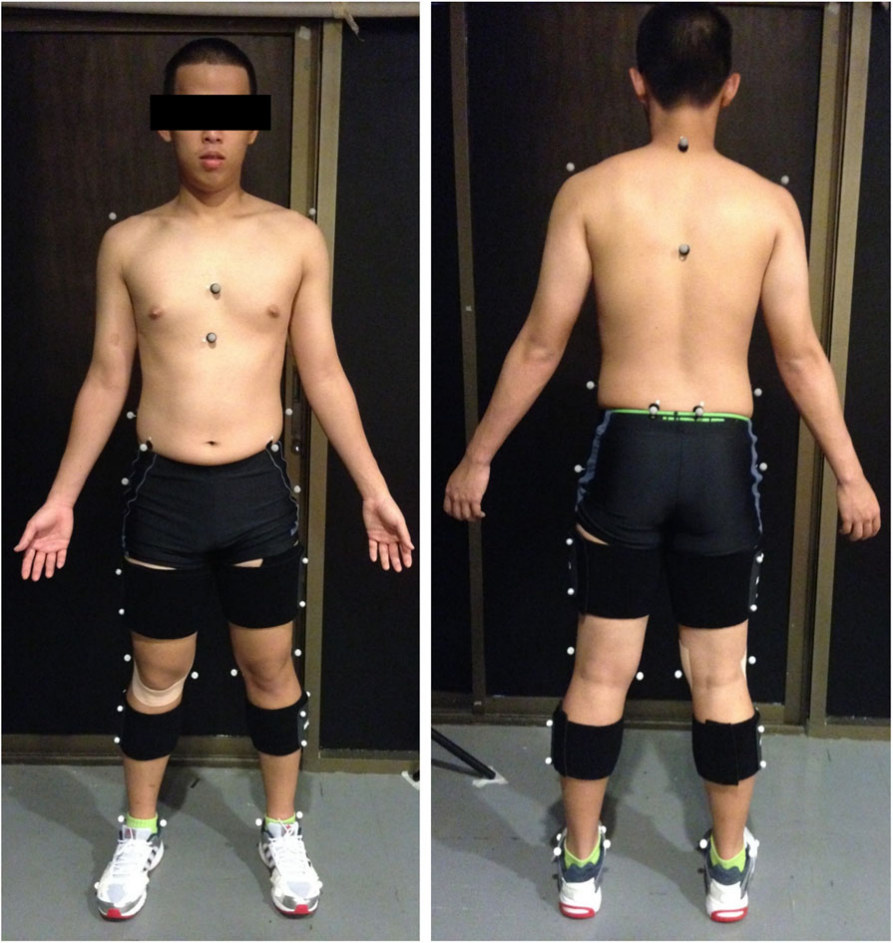
Marker placement

All data were collected with a 10-camera 3D motion analysis system (BTS Bioengineering Inc., Italy) at a sampling rate of 200 Hz and 2-force plates (model 9286BA, Kistler Inc., Switzerland) at a sampling rate of 1600 Hz. The 3-D LJMU model [14, 15] was applied during the data collection. 44 retorreflective markers were placed on the body at eight segments, including feet, upper and lower legs, pelvis and trunk (Fig. 1). The Visual 3D program (Version5; C-Motion, Germantown, MD) calculated kinematic and kinetic data of the knee joint. Marker trajectories and force were both filtered with a fourth-order low-pass Butterworth filter with a cutoff frequency of 20 Hz [5].

### Procedure

**Fig. 2:**
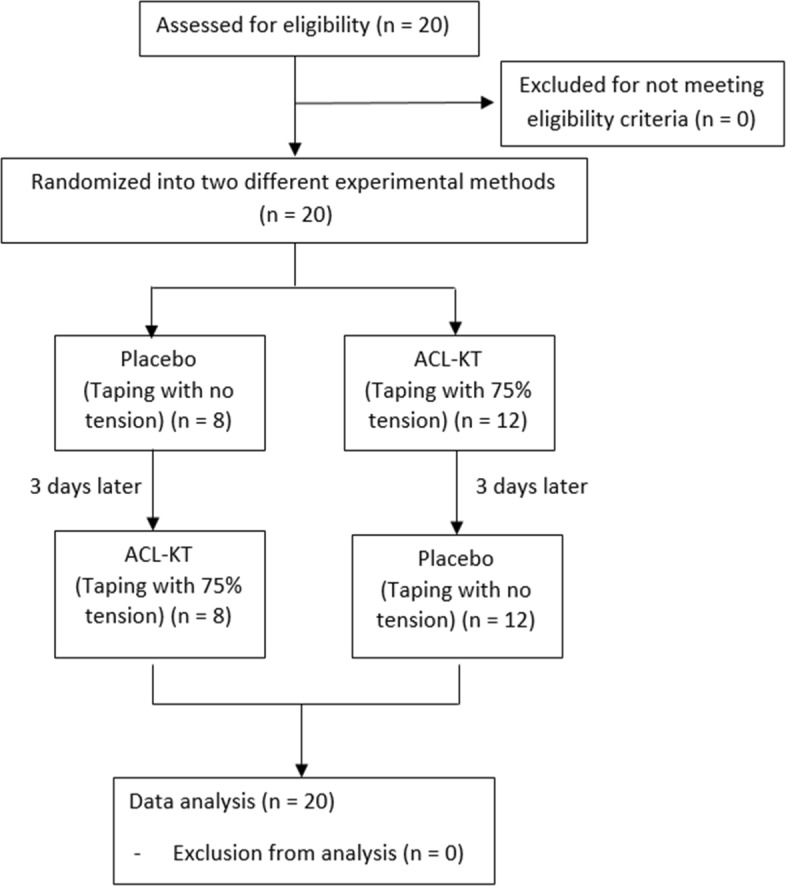
Flowchart of this study

Prior to the commencement of testing, each participant changed into shorts and a t-shirt and were provided with standardized running footwear (Duramo 5, Adidas, Germany), After retroreflective markers were placed upon the body, the participants were asked to perform a DVJ, The participants stood on top of a 31 cm box, dropped off the box, landed with knees bent and feet shoulder width apart before immediately performing a maximum vertical jump with both hands overhead and again landing with knees bent. The landing ground reaction forces (i.e., box landing, jump landing) of each foot were measured using two separate force plate platforms placed adjacent to each other. Three valid DVJ trials were collected for analysis. Each participant completed an ACL-KT condition and a placebo (PT) condition (described below) separated by three days in a counterbalanced order (Fig. 2). In order to ensure the consistency of taping between conditions, the same physiotherapist who was a certified KT practitioner applied all taping.

### Taping application

**Fig. 3:**
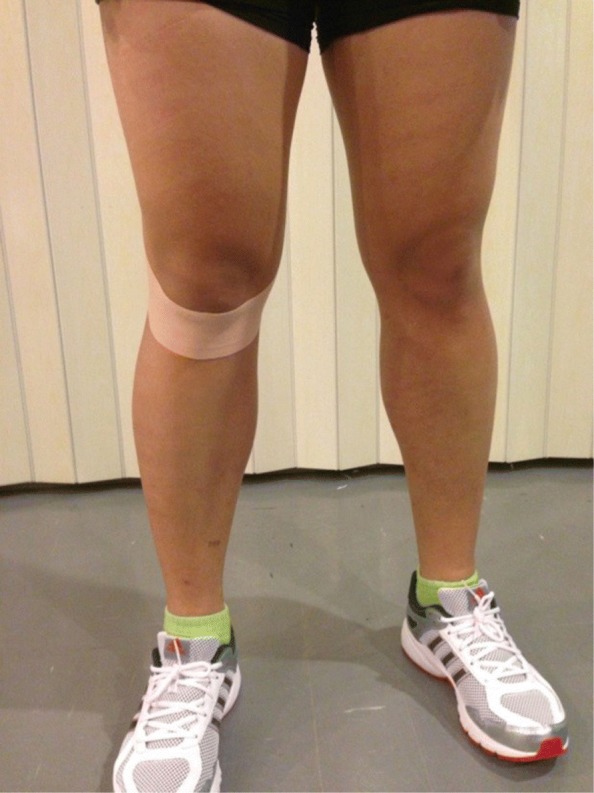
ACL-Kinesio Taping method

In the ACL-KT condition, participants had a standardized kinesio taping technique (for an ACL injury) applied to the leg. A 5 cm width of Kinesio tape (KinesioTex^®^, Japan) was cut into an I shape (30 cm) and was applied at the tibial tuberosity to the medial and lateral condyle of the femur with 75% of tension to limit anterior translation of the tibia [16] (Fig. 3). In the PT condition, the participants had the same technique applied with non-stretched Kinesio tape at 10% of resting tension. All participants were blinded to the experimental condition received.

### Data analysis

Knee joint angles at initial contact (IC) were selected for analysis. IC was defined as a vertical ground reaction force greater than 10 N [17, 18]. The knee joint angles and moments were selected from initial contact to take off from the first landing of each DVJ. The knee joint moments were calculated from inverse dynamics and represented as an external joint moment, e.g. an abduction moment is a moment generated by the environment that pushes the knee towards abduction. The dependent variables of this study were knee flexion and abduction angle at IC, peak knee angle and moment in three directions.

### Statistical analysis

The Shapiro-Wilk test was used to check normality of the outcome variables (SPSS v.17, SPSS Inc, Chicago). Comparison peak knee joint angles and moments, and knee joint angle at IC between conditions were analyzed using paired sample t-tests. The level of significance was set at *p*<0.05.

Although peak knee joint angles and moments, and knee joint angle at IC are critical variables to predict any effect of ACL-KT, the examination of these variables across the entire contact phase could also represent a biomechanical change during this important period, for example 100 ms after initial contact. Therefore, we used statistical parametric mapping (SPM) [19] to analyze the null hypothesis by statistically examining the whole joint biomechanics time-series. The resulting SPMt curve was used to indicate the significance of knee joint angles and moments at *p*<0.05. All SPM analyses were implemented using the open-source spm1d code (v.M0.1, www.spm1d.org) in Matlab (R2014a, 8.3.0.532, The Mathworks Inc, Natick, MA).

## Results

Knee Kinematics: A significant decrease in knee abduction angle at IC was found in the ACL-KT condition (F_19_ = 2.258, *p*=0.04). However, there were no significant differences in knee flexion angle at IC or peak knee flexion, abduction and rotation angle between the ACL-KT and PT conditions (*p*>0.05; Table 1).

**Table 1:** Mean (SD) of the knee joint angle at IC and peak knee joint angle between KT and PT conditions

Kinematic (degree)	KT	PT	Effect size	*p*-value
Knee flexion at IC (-)	-27.39 (10.96)	-30.96 (11.73)	0.28	0.337
Knee abduction (-)/ adduction (+) at IC	1.43 (2.12)	-1.24 (2.42)	1.06	0.036*
Peak knee flexion (-) /extension (+)	-96.22 (11.67)	-90.93 (11.08)	0.55	0.599
Peak knee abduction (-)/ adduction (+)	-2.00 (6.59)	-4.25 (5.93)	0.44	0.126
Peak knee external (-)/ internal rotation (+)	6.77 (8.96)	6.71 (8.81)	0.10	0.974

(Data are expressed as mean ± standard deviation. IC, initial contact. *Significant effect of taped, *p*<0.05)

**Fig. 4:**
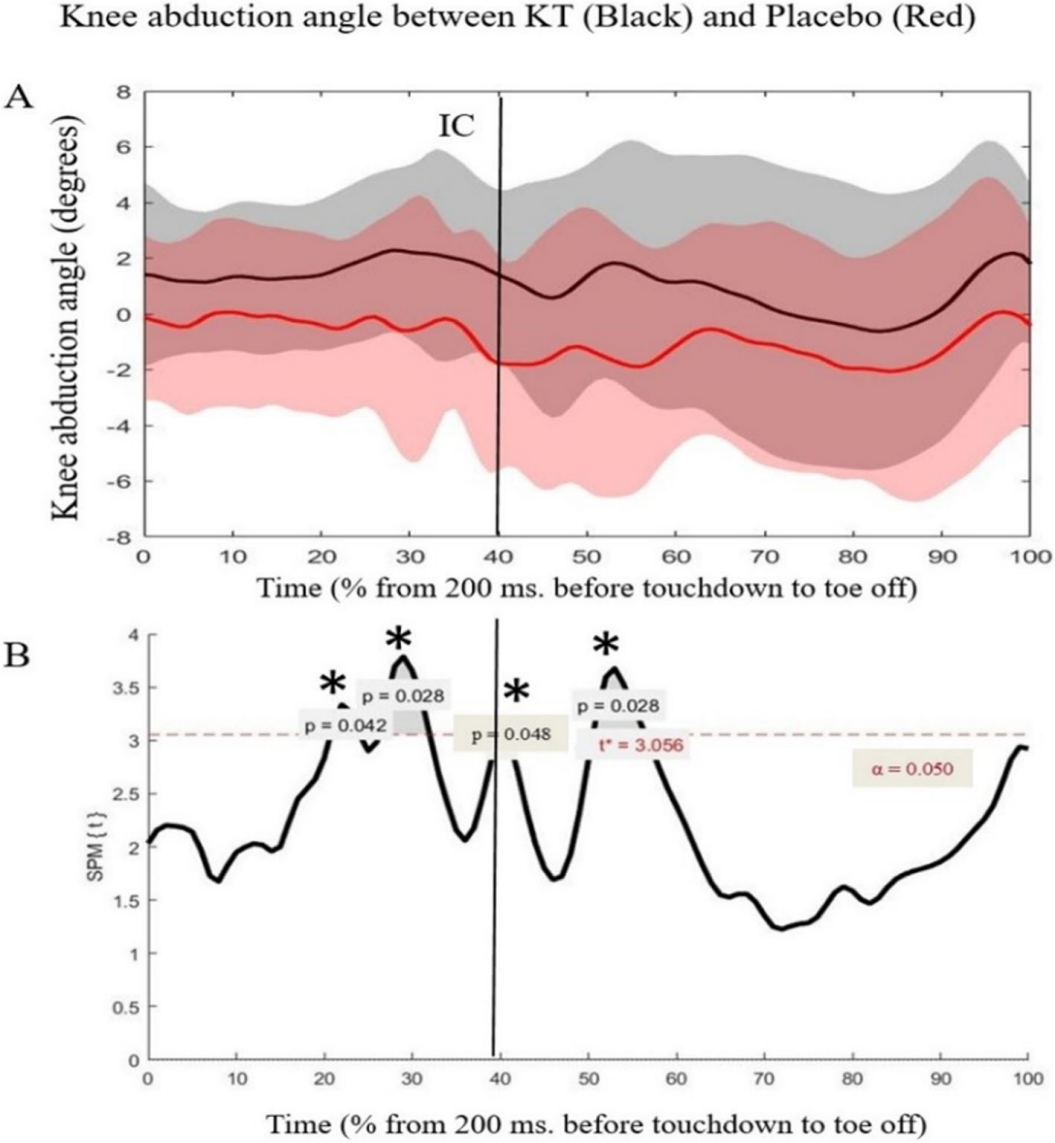
Knee abduction ankle between Kinesio tape (KT) and placebo condition **a**. Mean knee abduction angle between KT (Kinesio tape condition/Black), and PT (Placebo tape condition/Red) during landing phase. **b**. Statistical parametric maps for angle data. Mark (*) indicate a significant difference between taping conditions (p = 0.042, 0.028, 0.048 and 0.028, respectively). All curves are normalized from 200 ms before touchdown to toe off phase (%) of DVJ. IC is initial contact

The SPM analyses revealed a greater knee abduction angle in the PT compared with the ACL-KT condition at 20 to 55% contact time during the DVJ. Identically smooth random 1D data would cluster of this breath with a probability of *p* = 0.04, 0.03, 0.05 and 0.03, respectively (Fig. 4).

Knee Kinetics: There were no significant differences observed between the ACL-KT and PT conditions in any of the kinetic outcome measures (*p*>0.05; Table 2).

**Table 2:** Mean (SD) of the peak external knee joint moment between KT and PT conditions (Data are expressed as mean ± standard deviation)

Kinetic (N.m./Kg.)	KT	PT	Effect size	*p*-value
Peak knee flexion (+) /extension (-)	1.97 (0.33)	2.05 (0.51)	0.21	0.521
Peak knee abduction (+)/ adduction (-)	0.02 (0.40)	0.06 (0.37)	0.16	0.762
Peak knee external (+)/ internal rotation (-)	-0.10 (0.31)	-0.23 (0.32)	0.42	0.053

## Discussion

This study investigated the effects of ACL-KT on knee joint kinematics and kinetics during a DVJ in healthy subjects. Our results showed that ACL-KT, with 75% tension, had an effect of decreasing knee valgus angle during the 100 ms after IC. These findings are in agreement with previous researches that have indicated an ACL injury to typically occur within the first 100 ms after the foot touchdown [20, 21]. In the placebo condition, we used a non-stretchable KinesioTex^®^ tape, which has a significantly lower stress and load on the taping area compared with the KT condition [22]. Knee abduction angle at IC and peak knee abduction angle are directly associated with anterior tibial translation in male athletes during landing [4]. Previous study concludes that the prevention program should focus on a landing technique with knee flexion without valgus loading of the knee [21]. These results indicated that the ACL-KT might change behavior and pattern of a DVJ movement and that a DVJ has greater frontal plane movement [4, 23]. The key mechanism underpinning the use of kinesio tape is proposed to be the stimulated tactile stimulus on cutaneous mechanoreceptors, which enhances proprioception, joint position sense, and perception to avoid excessive movement [16]. However, there was no significant difference in other kinematic data because the tension force of ACL-KT was only applied between the tibial tuberosity to the medial and lateral condyle of the femur that may help to control anterior translation and valgus force. These outcomes are likely related to kinesio tape having highly elastic properties to allow a full normal range of motion whilst providing support.

The findings from the present investigation are in contrast to previous observations, which have reported no significant differences in peak knee joint angle and moment between KT and no tape (NT) during running [24] and no effect of KT to reduce knee shear force during ballet landing [25]. The Kinesio taping technique of previous studies were applied using a single Y strip applied with 25-50% tension over vastus medialis oblique and 75-100% tension laterally around the patella. In our study, we applied a standardized KT technique with 75% tension, typically used for ACL injury, which was applied directly to the tibia in an anteroposterior direction. This method may have helped to control the knee joint during the DVJ since different taping techniques and tension have an effect on the force and load around the taping area [22].

In this study, a comparative SPM analysis was used to examine the whole joint biomechanics time-series during the contact phase as the vector analysis only used one time point per se. The results showed that the ACL-KT had a significant effect on knee abduction angle before IC and 100 ms after IC. These findings point to the potential benefits of SPM analysis in examining the entire contact phase during a DVJ to assess ACL injury and possible interventions. Indeed, SPM analysis has become a gold standard due to its ability to standardize hypothesis testing of normalized data over time [26].

It is important to note there were limitations to our study. Firstly, we based our a priori analysis on knee valgus moment during running, however our aim was to determine knee valgus changes during a drop jump movment. Therefore, we carried out retrospective analysis using both the observed effect and variance in knee valgus between expreimental conditions, to confirm our study was adequately powered (post hoc sample size = 13). Despite incorporating a placebo condition (using tape without tension), we did not include a non-taped condition as a control. This may have helped clarify placebo effects, such as the influence of psychological and cutaneous mechanoreceptor activation. Secondly, our observations were related only to the relatively acute effects of KT and not the long-term effects, e.g. up to 24 hours after KT application, which remain unknown. Finally, the sample group in this study were male healthy participants, thus future research should test the effects of KT on symptomatic and/or female subjects [27].

## Conclusion

The application of standardized KT with 75% tension for ACL injury prevention revealed tangible changes in knee valgus angle at IC during a DVJ in healthy male participants. This justifies a need for continued research to reveal whether other biomechanical risk factors of ACL injury can be tangibly modified through KT application to support the evidence-based development of prevention programs.

## Data Availability

The datasets used and/or analysed during the current study are available from the corresponding author on reasonable request.

## Abbreviations

ACL: Anterior cruciate ligament
ACL-KT: Anterior cruciate ligament kinesio taping
DVJ: Drop vertical jump
IC: Initial contact
KT: Kinesio tape
PT: Placebo condition
SPM: Statistical parametric mapping
